# NMRbot: Python scripts enable high-throughput data collection on current Bruker BioSpin NMR spectrometers

**DOI:** 10.1007/s11306-012-0490-9

**Published:** 2013-01-04

**Authors:** Lawrence J. Clos, M. Fransisca Jofre, James J. Ellinger, William M. Westler, John L. Markley

**Affiliations:** National Magnetic Resonance Facility at Madison, Department of Biochemistry, University of Wisconsin-Madison, 433 Babcock Drive, Madison, WI 53706 USA

**Keywords:** NMR spectroscopy, Metabolomics, Compound screening, Automation, Data collection, Python scripting

## Abstract

To facilitate the high-throughput acquisition of nuclear magnetic resonance (NMR) experimental data on large sets of samples, we have developed a simple and straightforward automated methodology that capitalizes on recent advances in Bruker BioSpin NMR spectrometer hardware and software. Given the daunting challenge for non-NMR experts to collect quality spectra, our goal was to increase user accessibility, provide customized functionality, and improve the consistency and reliability of resultant data. This methodology, NMRbot, is encoded in a set of scripts written in the Python programming language accessible within the Bruker BioSpin *TopSpin*™ software. NMRbot improves automated data acquisition and offers novel tools for use in optimizing experimental parameters on the fly. This automated procedure has been successfully implemented for investigations in metabolomics, small-molecule library profiling, and protein–ligand titrations on four Bruker BioSpin NMR spectrometers at the National Magnetic Resonance Facility at Madison. The investigators reported benefits from ease of setup, improved spectral quality, convenient customizations, and overall time savings.

## Introduction

An increasing number of scientific investigations involve the analysis of large sample sets, often assembled in a range of divergent compositions. One of the best methods for atomic-level characterization of molecules and mixtures is solution-state nuclear magnetic resonance (NMR) spectroscopy (Maher et al. 2008; Shortridge et al. 2008; Xie et al. 2009). The unparalleled capabilities of NMR to acquire useful data, however, require a non-trivial level of expertise with an NMR spectrometer and familiarity with its underlying principles. To set up even the simplest one-dimensional experiments requires the spectrometer user to spend several minutes optimizing several hardware and software parameters. For example, the spectrometer probe must be “tuned and matched” for each new sample placed in the spectrometer to maximize the efficiency of radio-frequency (RF) signals sent and received from that sample. In addition, the magnetic field passing through the sample needs to be made as homogeneous as possible in order to optimize spectral lineshapes. This process, called shimming, is achieved by adjusting the electrical current in a multitude of “shim” coils directly adjacent to the sample. Also, the pulse program that dictates which nuclei are probed for each experiment contains radio frequency (RF) “pulses” that need to be calibrated for optimal signal-to-noise (S/N). Other parameters, such as the range of frequencies to sample (spectral-width, or SW), are difficult to determine a priori, and must be manually deduced for subsequent data collections. Hence, experiment setups for a large set of samples can potentially consume a significant portion of an investigator’s time and effort.

In recent years Bruker BioSpin has introduced several hardware and software products that soften the requirements for user technical expertise and promote high-throughput NMR spectroscopy. Hardware accessories for automated probe tuning and matching (ATM) and sample-tube changers (SampleJet™) provide users the convenience of manipulating the probe or sample, respectively, from the newest versions of *TopSpin*™, Bruker’s software for NMR data acquisition and analysis (Soininen et al. 2009). *TopSpin* provides the interface for these hardware accessories, as well as automated procedures for sample shimming, pulse calibrations, and receiver gain optimization. All these features are especially useful for spectrometers that can be remotely operated. *TopSpin* includes a legacy software suite, ICON-NMR, for high-throughput data acquisition that incorporates many of the software features described previously.

In attempting to perform several studies at the National Magnetic Resonance Facility at Madison (NMRFAM) involving large sample sets (e.g. metabolomics, crude extracts, small-molecule libraries, protein–ligand screening), we encountered limitations to ICON-NMR that hinder accessibility, preclude sample set heterogeneity, and limit the quality of acquired data. The specific limitations in ICON-NMR include: a restrictive interface with access separate from *TopSpin*; a complicated and inflexible menu system for sample information entry; reduced performance of automated sample shimming; difficulty in accommodating different solvents in the same sample set; and an inability to adapt experiment parameters to each sample. These limitations provided the impetus to develop a more straightforward, intuitive, high-throughput methodology for automated data acquisition across diverse sample sets. Our goals were to simplify the setup procedure for data acquisition, provide easily customizable functionality, and improve the quality of data acquired over what was previously obtained from ICON-NMR.

## Methods

Automated, high-throughput NMR data collection for large sample sets first requires access to a spectrometer equipped with the automated hardware and software features described in the introduction. For our purposes, we developed and tested this new methodology on four Bruker BioSpin NMR spectrometers at NMRFAM; a 500 MHz Avance III with 5 mm triple resonance cryoprobe, a 600 MHz Avance III with 5 mm quadruple resonance cryoprobe, a 600 MHz Avance III with 1.7 mm triple resonance cryoprobe, and a 700 MHz Avance III with 5 mm quadruple resonance cryoprobe. Each spectrometer was equipped with SampleJet and ATM accessories, running *TopSpin* v. 3.0 under CentOS 5. To develop our methodology we utilized the Python programming language (Conway 1995) interpreter recently added in version 2.0 of *TopSpin*. It should be noted that we began development of NMRbot using *TopSpin* v. 2.0, hence NMRbot is backward compatible with this earlier version of *TopSpin*. The interpreter currently accepts functions from the Python v. 2.7.3 standard library and a number of modules designed by Bruker to access specific spectrometer functions. Methodology development focused on three areas: (1) design of an intuitive and flexible user interface for entry of sample information and experimental parameters, (2) automated operation of the spectrometer and cursory spectral analysis, and (3) sample data tracking and error handling.

**Fig. 1:**
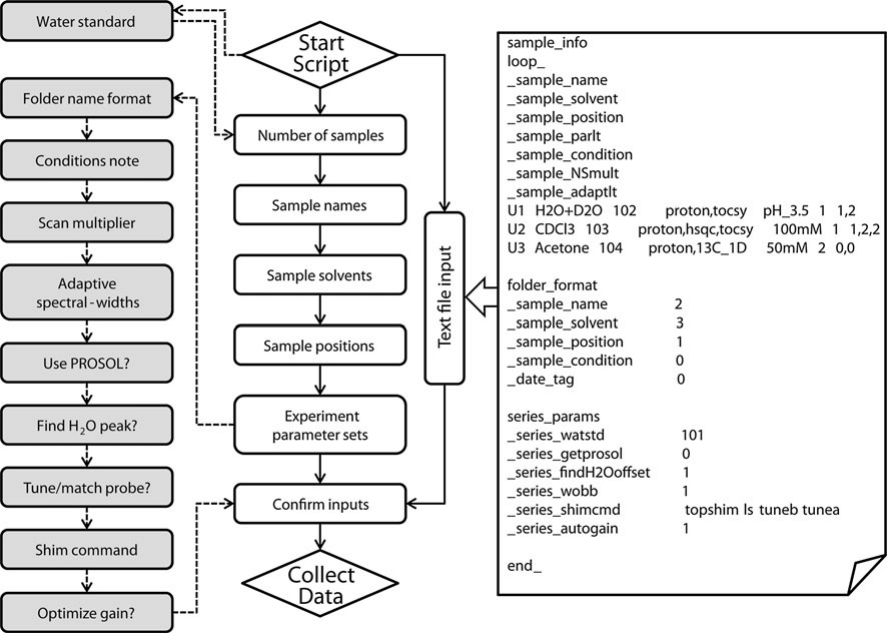
NMRbot sample and experiment parameter input methods. Flowchart of the *Setup Wizard* (*left*) user interface showing start and end points (*diamonds*), requisite inputs for manual path (*white boxes*), optional manual inputs (*shaded boxes*), and optional course for text file input (*right*). The expansion shows an example of an input text file in STAR format (Hall 1991) containing relevant sample and experiment parameters

To provide users with a more straightforward and flexible interface to setup automated data acquisition, we developed two approaches to input sample and experiment information. Execution of the Python script starts the Setup Wizard that provides access to both approaches (Fig. 1). As such, the first window of the Setup Wizard asks users to select between manual or text file modes for input. The manual input mode involves a series of input windows prompting the user for pertinent details (Fig. 1, left). A Python function was developed for each window, designed to verify user input or identify input errors. Each window allows the user to step forward or backward in the setup process. Alternatively, the text file input mode circumvents the manual input windows and prompts the user to enter the name of a text file that contains sample and experiment details (Fig. 1, right). The details in this file must be enumerated in the Self-defining Text Archive and Retrieval (STAR) format (Hall 1991), with sample specific information denoted separately from optional folder format and series parameters. A Python function to decode this type of text file was designed to validate the inputs and identify potential errors before acquisition begins. For both input modes, requisite input details are the number of samples, names, solvents, rack position, and experiment parameter set names (Fig. 1, white boxes in center). For experiment details, our method relies on predefined experiment parameter sets, a convention employed by Bruker to easily recall all spectrometer parameters for a specific experiment. Any number of available optional inputs, as described below, for customized optimization and operation of the spectrometer can be easily appended in the initialization script.

The optional inputs are described here in the order they appear in the manual input mode of the Setup Wizard (Fig. 1, left). The option to include a water standard sample (90 % H_2_O, 10 % D_2_O) is offered, and is the first to appear to differentiate from other samples in the series. This feature uses a “water” standard sample to establish optimal three-dimensional sample shims with *TopShim*, the automated shimming routine in *TopSpin*. After the requisite sample set information described above is collected, further optional inputs are presented. The first two provide simple bookkeeping preferences for sample folder name format and sample condition notes. Subsequent input options allow the user to tailor certain acquisition parameters for specific samples or the entire set. Ideal for low concentration samples in the sample series, an option is offered to multiply the number of scans (NS) for every NMR experiment of that sample by a given factor. The next optional input allows the user to define which one-dimensional experiments should be used to identify the range of observable sample peaks, as well as to define any subsequent experiments that should have their SW parameters adapted to this range along with a change of the spectrum center (offset) corresponding to the center of this range. Another option asks if the user wants common RF pulse parameters (length and power) to be automatically loaded from the “PROSOL” table, another Bruker convention in *TopSpin*, rather than using those in the loaded parameter set. If a sample in the series has a large solvent peak such as H_2_O, the user can opt to have the position of that peak automatically determined and entered as the offset. The next option can be selected to automatically tune and match the spectrometer for each new sample in the series. Another optional input allows the user to define the desired *TopShim* command used to optimize the shims for each sample. A final optional input allows the user to toggle the use of the “rga” function in *TopSpin* that automatically optimizes the receiver gain parameter for each experiment. If setup details are manually entered into the Setup Wizard, those details are compiled and output as a STAR formatted text file. This file, or altered versions thereof, can be used as text file input to future executions of NMRbot. Once all information has been input into the Setup Wizard, either manually or by text file, a final window allows the user to review the parameters entered. Upon confirmation, automated data acquisition begins.

We have developed several Python functions to automate the process of data acquisition and analysis. Many of these functions rely on core spectrometer interface functions included in the *TopSpin* Python interpreter. The most commonly utilized function in our methodology passes commands directly to the command line of *TopSpin*. This allows our methodology to largely follow the standard series of commands for manual operation of the spectrometer. In this way samples are inserted and shimmed, a deuterium lock is established, the probe is tuned and matched, sample data folders are created, and experiment parameters sets are loaded. These steps can be circumvented or modified by any optional inputs submitted by the user, such as skipping the tune/match step, changing experiment parameters from PROSOL or NS options, or optimizing the receiver gain. If the user opts to find the solvent peak for a sample, a one-dimensional ^1^H NMR experiment is loaded separately and a spectrum is acquired, followed by automated peak-picking and peak analysis to determine the position of the large solvent peak. This value is then passed internally to all experiments loaded for that sample to define the ^1^H dimension offset. The function developed for peak list analysis is also used to determine optimum SW (and corresponding offset in the center of this range) for any spectral dimension, if the user included this option. This information is passed along to any experiments flagged by the user for adapted SW. As with the Setup Wizard input functions, the automated data acquisition functions are designed to validate each step of data acquisition; if an error is encountered, it is logged and the acquisition proceeds to the next experiment in the list.

During development and testing, we found it advantageous to audit the progress of sample setups and automated data acquisition. This feature provides an accounting of each step of the method with real-time updates of the software’s activity displayed in the terminal window associated with *TopSpin*. Also, these updates are appended to an audit text file output in each sample’s data directory, and all updates of the sample series are likewise output to the user’s experiment directory. Any errors encountered during automated data acquisition are also included in these audit tracks. We made every effort to design the acquisition functions so that they would continue on to the next procedure upon error detection.

All the functions are combined in a single script file, named FAM_Tools.py, and placed in the directory <*TopSpin* *home*>/exp/stan/nmr/py/user. The same directory contains a short script file, named NMRbot.py, which begins the initial process of information collection from the user. Every session is invoked by typing the name of the short script file in the command line of *TopSpin*.

For direct comparison, identical NMR experiments were acquired on several complex mixture samples using the two automated methods, ICON-NMR and NMRbot. The ^1^H 1D, ^13^C 1D and ^1^H-^13^C 2D HSQC experiments used the same parameter sets and shimming routine. An additional 2D HSQC was acquired with NMRbot, employing the adaptive spectral-width feature to automatically determine the optimal ^13^C dimension SW parameter using peaks observed in the ^13^C 1D experiment.

## Results and discussion

The NMRbot method was able to reproduce the basic behavior of ICON-NMR for automated data acquisition on a series of samples. For example, the time to acquire the data using the ICON-NMR and NMRbot methods was comparable, however the setup of NMRbot required less time from the user and several novel NMRbot methods did add small amounts of time in certain circumstances, as described below. The setup time saving for NMRbot as compared to ICON-NMR varied between about 5 min (for manual input) and 20 min (for text file input). User feedback during development indicated that the two NMRbot input methods, especially the text file input method, were found to be more accessible and straightforward than the ICON-NMR interface. Users attributed this to two main differences of the input interface: (1) the successive prompts of the NMRbot Setup Wizard present the user with all pertinent setup variables, reducing the chance of a missed input and distilling the functional complexity of the interface; (2) eliminating the requirement for familiarity with drop-down menus and other non-obvious input methods, as are employed in ICON-NMR, thus lowering the learning-curve to use NMRbot and reducing the cognitive complexity of the interface. In fact, the NMRbot text file input mode obviates most of the Setup Wizard interface.

**Fig. 2:**
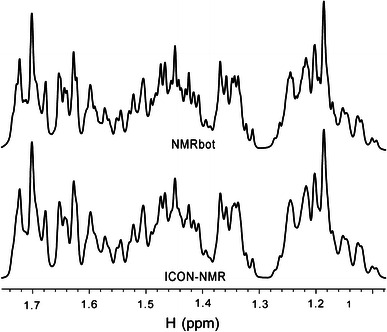
^1^H NMR spectra of a complex mixture collected by two automated methods, (*bottom*) ICON-NMR and (*top*) NMRbot, which use the same automated shimming method (*TopShim*). The spectrum shimmed under the NMRbot protocol shows slightly better resolution

Several important differences were noted in the performance of the two automated methodologies (see Table 1). First, ICON-NMR displayed difficulty in automatically determining optimum solvent lock parameters for samples using different solvents than proceeding ones in the series. This difficulty was not encountered with NMRbot. Presumably, ICON-NMR uses the same software protocol as NMRbot, which is to call the “lock” procedure as is done manually, so this difference in performance occurred for reasons unknown. Second, the automatic shimming protocol produced better lineshapes in NMRbot. The spectra in Fig. 2 show a clear improvement in resolution for the NMRbot acquired spectrum, and hence allowed more complete analysis of this sample. Again, this difference in performance is inexplicable given that both methods rely on the *TopShim* procedure in *TopSpin*. These performance limitations for ICON-NMR impacted the quality of acquired spectra, potentially wasting spectrometer time and requiring manual reacquisition. Indeed, one user reported as many as 50 % of spectra as “unusable” when acquired with ICON-NMR. All spectra collected thus far with the fully developed NMRbot method have met quality criteria for each user.

**Table 1:** Qualitative assessment of NMRbot features as compared to ICON-NMR

Feature	NMRbot	ICON-NMR	ΔT (min)
User interface	✩✩✩	✩	−5, −20^a^
Sample shimming	✩✩✩	✩✩	–
Probe tuning	✩	✩	–
Sample handling	✩	✩	–
Adapted spectral-width	✩	X	–
Scan multiplier	✩	X	–
Optimize offset	✩	X	<1
Optimize gain	✩	X	<0.5
H_2_O std. shimming	✩	X	<10^b^
Text file audit trail	✩	X	<1

Stars in the method columns indicate the presence of a feature and, if applicable, the number of stars indicates feature performance as determined by NMRbot user feedback. An “X” indicates the absence of a feature. The time difference (ΔT) column indicates any NMRbot feature time difference as compared to ICON-NMR

^a^Time savings from NMRbot manual or text file input methods

^b^Water standard sample shimming is lengthy, but can reduce time for later procedures (see text)

**Fig. 3:**
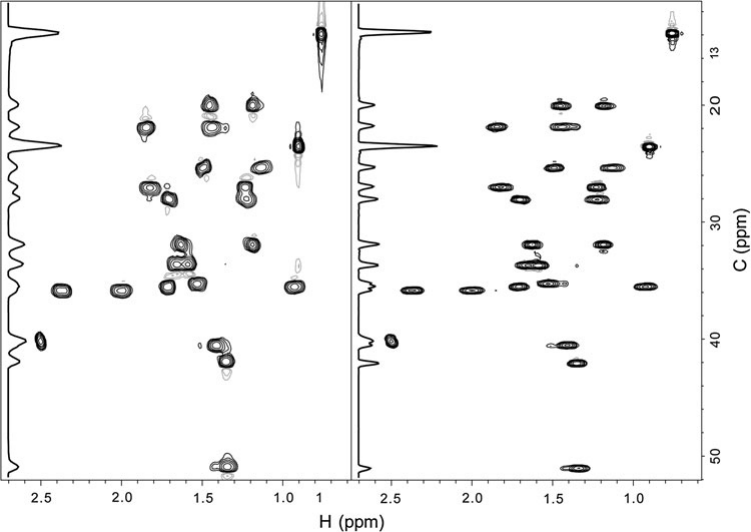
Improvements in 2D ^1^H- ^13^C HSQC spectral quality due to adaptive spectral-width. *Black* contours show positive spectral intensity, while *grey* contours show negative intensity. The 2D HSQC spectrum on the* left* was collected with general acquisition parameters (i.e. ^13^C-dimension SW parameter of 200 ppm). The spectrum on the* right* was collected using the same acquisition parameters, except for a 50 % reduction in spectral-width and a corresponding change in ^13^C-offset (*dimension center*) as determined by automated analysis of a 1D ^13^C spectrum acquired previously in the experiment set. The gains in ^13^C resolution and decoupling efficiency are* highlighted* by the trace along the *left edge* of each spectrum

Several features developed for NMRbot provided other distinct advantages over ICON-NMR. The option to initially perform three-dimensional shimming on a water standard sample did add up to 10 additional minutes to the overall acquisition time of NMRbot, but with a slight reduction in shim times and improved resolution for subsequent samples in the series. Other optional inputs allowed specific parameters to be automatically determined and modified on-the-fly, increasing data quality and consistency. These features also preclude the need to create separate parameter sets for specific samples. This enables NMRbot to facilitate study of diverse sample sets. For example, the input NS multiple improved the S/N for all data acquired on low concentration samples using the same parameter sets as other more concentrated samples in the series. For samples with large solvent peaks that needed to be suppressed by presaturation, the ability of NMRbot to automatically determine the offset allowed a general parameter set to be used and did not require the investigator’s time to predetermine the offset. The method to automatically determine the optimum offset, however, did add up to 60 s to the overall method per sample. Moreover, use of the adaptive spectral-width feature enhanced data resolution and promoted more effective decoupling in certain experiments. This advantage is shown in Fig. 3, which compares spectra initiated from the same parameter set, one with default settings and the other with the adaptive spectral-width feature enabled for the ^13^C dimension. The time required for automated peaklist analysis to determine adapted spectra-width parameters was negligible (1–2 s), and slightly increased total acquisition time when applied to the direct dimension of subsequent experiments.

The creation of audit trails for each sample and the entire sample series provides investigators with an accounting of all spectrometer activity and a means of validating data acquisition procedures. This feature is also useful for discerning the time and nature of acquisition errors, helping NMRbot users and developers alike. Other convenience features, such as folder name format, condition notes, and automated spectrum title details, aid users in organizing and tracking acquired data.

## Concluding remarks

Our motivation for developing NMRbot as a custom-built application arose from perceived limitations in the available automated data acquisition software for Bruker NMR spectrometers. Its initial development was meant to bypass these limitations, but we quickly determined that other improvements would assist the needs of investigators at NMRFAM. NMRbot provides an accessible, robust, time-saving setup interface for spectrometer users of all stripes. Optional features expand the functionality of automated acquisition and further save investigator time by automating the determination of several parameters that enhance data quality and consistency. This methodology currently stands as an alternative to Bruker’s ICON-NMR. In future releases of NMRbot, we plan to include features such as series completion time calculations and a run-time interface for more user control during acquisition.

The Python scripts for NMRbot are currently available from the NMRFAM website (www.nmrfam.wisc.edu/software/nmrbot/), along with simple installation and usage instructions.
